# Soft tissue attachment of human gingival fibroblasts to titanium dioxide nanotubes compared to commercially pure titanium and its alloys: a systematic review

**DOI:** 10.1038/s41405-025-00293-0

**Published:** 2025-06-18

**Authors:** Sara Baraka, Anu Sam, Marta Krysmann, Neil Cook, Waqar Ahmed, Fadi Barrak

**Affiliations:** 1 School of Medicine and Dentistry, University of Central Lancashire, Lancashire, UK; 2 School of Mathematics and Physics, College of Health and Science, University of Lincoln, Lincoln, UK; 3 VSSAcademy, London, UK

**Keywords:** Dental materials, Dental biomaterials

## Abstract

**Objective:**

This systematic review was conducted to evaluate the attachment of human gingival fibroblasts (HGFs) of the soft tissue, to titanium dioxide nanotubes (TNTs) compared to commercially pure titanium (cp-Ti) and its alloys, in in-vitro studies. It is postulated that the nanotopography of the TNTs provide cells with a biomimetic environment, allowing HGFs to form more focal adhesion (FA) attachment sites at the tubule edges.

**Method:**

A comprehensive literature search was conducted on MEDLINE, DOSS, EMBASE and Google Scholar from January 2012 to January 2022. The identified studies were screened based on titles and abstracts for inclusion criteria. The relevant studies underwent data extraction. The risk of bias was assessed through the Office of Health Assessment and Translation (OHAT) tool.

**Results:**

This systematic review included four studies evaluating cell proliferation, protein expression, gene expression and cell morphology of HGFs evocative of stronger and mature soft tissue attachment. A significant increase in the cell proliferation at TNTs compared to cp-Ti, at day 7 for three studies and at day 14, for one study was evident. In addition, a significant increase in the type 1 collagen protein expression at TNTs compared to cp-Ti, at day 6 for one study and day 7 for two studies. Enhanced cellular extensions from HGFs attached onto TNTs, compared to cp-Ti was observed in all four studies. All the primary effects evaluated suggest the formation of better interlaced fibers giving a stronger adhesion than the parallel ones which is the most relevant outcome of this research.

**Conclusion:**

HGFs showed enhanced contact guidance onto TNTs but a true biological attachment was not confirmed. This review involved invitro studies which lack methodological rigor to compare among studies, lack information and have small sample sizes limiting effectiveness of parametric tests. The results may be unpredictable when translated to in-vivo studies mainly affected by confounding factors. Further research is needed to determine the precise mechanism of mechanical attachment between the soft tissue and the transmucosal surfaces.

## Introduction

**Fig. 1:**
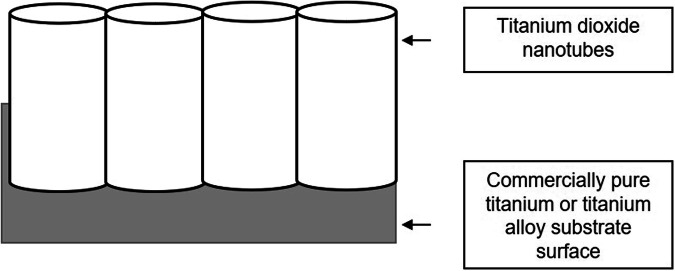
TNTs superimposed on a titanium surface [22]. Diagram representing the cylindrical appearance of titanium nanotubes with the base attached on the implant body composed of commercially pure titanium, or its alloys.

In implant restoration, establishing a robust soft tissue seal is essential for preserving peri-implant health and long-term success as it creates a protective barrier, prevents bacterial invasion and the apical migration of the junctional epithelium, and helps minimize bone loss [1]. The pursuit of an optimal transmucosal implant surface with enhanced human gingival fibroblast (HGFs) attachment, has captured the attention of researchers, clinicians, and manufacturers [2–14]. Within nanotechnology, the focus has been on nano-scale surface modification of titanium implant surfaces with controlled nanostructures [2, 3, 5–9, 12, 13]. Titanium dioxide nanotubes (TNTs) are tiny structures resembling nano-scale test-tubes with a closed bottom and an open top, aligned vertically and superimposed on a titanium surface (Fig. 1) [2, 7, 9, 13, 15–19].

TNTs have a large surface area, good corrosion resistance and excellent biocompatibility [5, 9, 20, 21]. Adhesion of TNTs to the titanium substrate surface is essential to ensure a mechanical interlocking and stability [17–19, 22].

This nanotopography provides a biomimetic environment, enhances bioactivity, facilitates the attachment of cells, and encourages integration with the host tissues at the nanotubes edges [23].

Self-organised arrays of TNTs, using electrochemical anodization have been investigated [24]. After ultrasonic cleaning, a direct current voltage is then applied to the titanium substrates causing anodic oxidation in a fluoride-based electrolyte to obtain TNTs [9, 22]. After annealing at 500 °C for 2 to 4 hours in the air and being ultrasonically cleaned, the TNTs were prepared [25]. This relatively low-cost technique gives good control of the surface morphology [21]. The *“groove like texture”* at a nanoscale level on the TNTs enhances early cell adhesion and proliferation via contact guidance [7].

Studies have shown that after biomaterials are implanted into the body, they first undergo hydration with water molecules rapidly, and then small molecular proteins first adhere to the surface of the implant, followed by the exchange and adsorption of protein molecules, i.e., the Vroman effect [26]. Hence, it is crucial to reduce surface potential and increase the hydrophilicity of the implant surface. This is also called as surface wettability. As the physiologic liquids viz, blood and serum rapidly envelop the implant surface, they allow rapid specific germ cell enticement onto the implant surface, in this case, the fibroblasts.

Recent research has demonstrated HGF cell alignment along the direction of nanostructures including nano-grooves and nano-pores suggesting a strong mechanical stimulation postulated that HGFs may migrate towards the titanium abutment surface and then deposit the ECM or attach directly to the blood-derived fibrin or proliferate, migrate, and then attach onto the abutment surface [8, 27–30]. The HGF cells secrete the extracellular matrix (ECM), which is a vital physical scaffold and mediator of cell adhesion [10, 27]. HGFs secrete various ECM proteins including the structural protein called collagen type 1 (COL-1) and the adhesive protein called fibronectin [10]. Subsequently, these proteins bind to focal adhesions, including integrins, which are transmembrane receptors on the surface of HGFs, mediating signal transduction and promoting soft tissue attachment [31].

This systematic review was conducted to determine if HGFs have an enhanced soft tissue attachment to TNTs compared to cp-Ti or Ti-Al6-V4, in-vitro.

## Materials and methods

### Search strategy

A systematic review was conducted across three electronic databases. MEDLINE and Dentistry and Oral Sciences Source (DOSS) were searched using EBSCO Host platform on 8/2/2023. EMBASE was searched using the Ovid interface on 8/2/2023. A search limit of studies published between January 2012 to 2022 was applied, as the evidence base from recently published articles is current, up-to-date, relevant and has the potential to bridge research gaps. These were supplemented with citation chaining of the references in existing systematic reviews [27, 32, 33]. Google Scholar was used to identify any studies not found from the databases used. Key words and MESH (or equivalent) terms were refined and adapted accordingly for each database used. Truncation (*) was used to maximise search results. The search terms were developed according to the PICO framework [34], detailed in Table 1.

**Table 1:** PICO framework

Population	*Human gingival fibroblasts*
Intervention	Titania nanotubes
Comparator	Commercially pure titanium or titanium alloy surface
Outcome	Soft tissue attachment

The search strategy for MEDLINE is presented in Index 1.

The full text was obtained for the titles and abstracts that fulfilled the inclusion criteria or where the eligibility was unclear. These were screened and the relevance assessed using the following.

#### Inclusion criteria

**Population** - HGFs from human donors (primary cells) or commercially available cell collection (cell line).

**Intervention** - TNTs fabricated using electrochemical anodization on a cp-Ti or Ti-Al6-V4 substrate surface. The TNTs used were untreated. If the study used both untreated and treated TNTs, only the untreated TNTs were included in data extraction and analysis.

**Comparator** - Standard machined/smooth/turned/polished cp-Ti or Ti-Al6-V4 surface materials. If the study reported more than one type of comparator/control, only the machined/smooth/turned/polished cp-Ti or Ti-Al6-V4 surface were included.

**Outcome Measure** - Cell proliferation measured by colorimetric assays including CCK-8, MTT or MTS to demonstrate cell viability. The expression or secretion of proteins by HGFs was measured using ELISA. The expression or secretion of one or more of the following proteins must be demonstrated: collagen type 1 and/or fibronectin. Additional outcome measures may include gene expression levels and cell morphology. If gene expression was included as a supplementary outcome, it should have been measured using RT-qPCR. Cell morphology must be evaluated using SEM.

**Study Design** - In-vitro studies.

**Context** - Only full reports and studies available online prior to publication were considered. Only English language publications were included.

The following **EXCLUSION CRITERIA** was applied:

**Population** - Any cell other than HGFs or HGFs co-cultured with other cells. Gingival fibroblasts were obtained from a non-human donor.

**Intervention** - Other nanostructures such as nanopores, nanowires, nanorods, nanobelts, nanoribbons, nanofibers, and nanoparticles. Treated TNTs using thermal hydrogenation, doping with antibiotics or nanoparticles.

**Comparator** - Surfaces not machined/smooth/turned/polished on cp-Ti or its alloys. 3D printer using laser technology.

**Outcome Measure** - Did not measure cell proliferation and protein expression. Did not demonstrate collagen type 1 and fibronectin expression.

**Study Design** - incorporated co-culture with other cell lines, in-vivo studies, 3D human or animal tissue models, clinical studies, and systematic reviews.

**Context** - Unpublished and grey literature. Non-English language. Abstracts of reports at the pre-result stage. Not primary research.

### Risk of bias assessment for included studies

The risk of bias was assessed for each included studies and each outcome measure separately, using the OHAT tool. This tool is recommended by the National Health and Medical Research Council for Systemic review or Meta-Analyses of in vitro studies as the following domains are assessed; rationale of the study, samples, randomization, blinding, procedures, reported outcomes, discussion evaluation and other bias [35]. The criteria used to assess the risk of bias is shown in Table 2.

**Table 2:** Risk of bias assessment adapted from the OHAT tool [48, 49]

Risk of Bias Criteria	Article 1	Article 2	Article 3	Article 4
Randomisation				
Allocation concealment				
Identical experimental conditions				
Blinding of researchers during study				
Incomplete outcome data				
Exposure characterisation				
Blinding of outcome assessors				
Outcome reporting				
Other sources of bias				

The following key was used in conjunction with the OHAT tool.

**Key**

– Definitely high risk of bias

NR Not reported.

- Probably high risk of bias

+ Probably low risk of bias

++ Definitely low risk of bias.

## Results

**Fig. 2:**
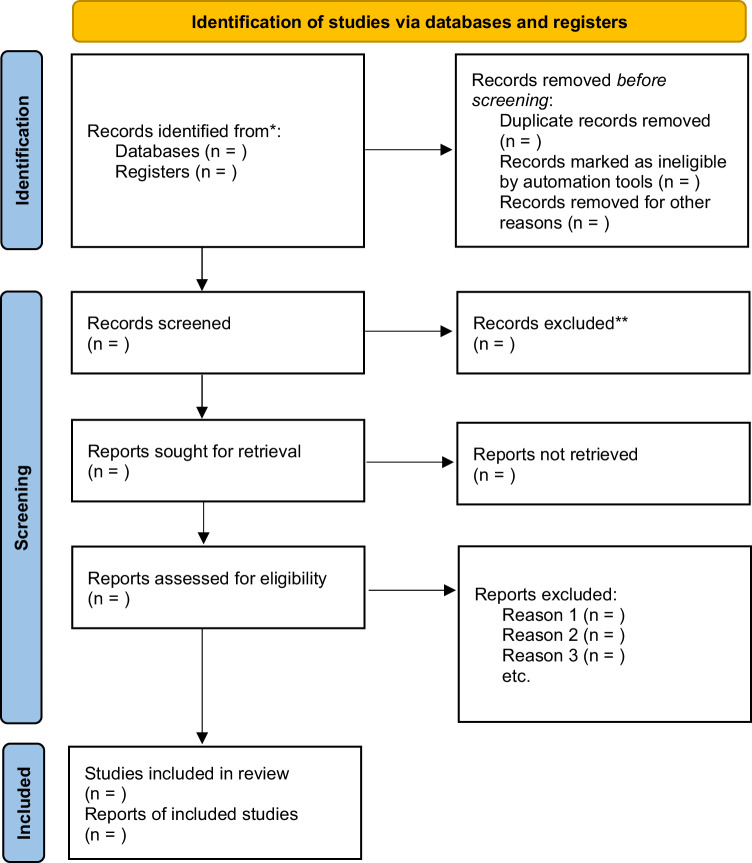
PRISMA workflow for the selection of eligible studies [84]. Flow chart describing the process of study selection for the systematic review.

Electronic and hand searches identified 589 citations of which 198 were duplicates. The remaining 391 citations were screened for inclusion. Their titles and abstracts were assessed. 378 studies were excluded at this stage. This left 13 studies for which the full text was obtained. Based on the exclusion criteria, 9 studies were excluded; 3 did not include the appropriate population [18, 36, 37]; 2 did not examine the appropriate intervention [10, 38]; 1 did not include the appropriate comparator [39]; and 3 did not examine the appropriate outcomes measures [2, 19, 40]. Four studies were included in the systematic review. The PRISMA flowchart (Fig. 2) summarises the study selection process [41, 42].

The study characteristics are presented in Table 1 and the findings for primary and secondary outcome measures are presented in Tables 2 and 3, respectively.

**Table 3:** Study characteristics

Author and year	Guida et al. [43]	Liu et al. [44]	Chen and colleagues	
			Wang et al. [25]	Cao et al. [1]
Study design	In-vitro	In-vitro	In-vitro	In-vitro
Location	Italy	China	China	China
Study funding	P.H.I. (San Vittore, Italy) provided the titanium samples.	Natural Science Foundation of China. Natural Science Foundation of China for young scholars.	National Natural Science Foundation of China.	National Natural Science Foundation of China
Population	HGFs	HGFs	HGFs	HGFs
Source of cell	Human biopsy (male and female donors)	Cell line from cell bank (ATCC) from male patient	Cell line from cell bank (ATCC) from male patient	Cell line from cell bank (ATCC) from male patient
Type of titanium sample	10 mm x 10 mm x 1mm plates	2 cm x 3 cm x 0.1 mm sheets	10 mm x 10 mm x 0.2 mm discs	1 cm x 1 cm sheets
Comparator	Turned cp-Ti	Polished cp-Ti	Smooth cp-Ti	Smooth cp-Ti
Intervention	TNTs	TNTs	TNTs	TNTs
Pre-treatment of Ti	None	mechanically polished and sonicated in acetone for 30 minutes	ultrasonically cleaned	None
Process of anodization	aqueous solution of 1 M sulphuric acid and 0.15% hydrofluoric acid at 20 V at room temperature	at 25 V for 1 hour at 500 mV seconds-1,in an electrolyte 1,2,3-propanetriol, NH4 F (1.0% wt), and H2 O (15% vol)	at 50 V for 15 min in an electrolyte composed of ethylene glycol containing 0.5 wt% ammonium fluoride (NH 4 F) and 10 vol% deionized water	(50 V, 15 min) in ethylene glycol electrolyte (containing 10 vol% deionized water and 0.5 wt% ammonium fluoride)
Post anodization treatment	rinsed in distilled water and subsequently in acetone, and finally dried with nitrogen stream.	annealed in air at 450°C for 4 hours	Annealed at 500 °C for 2 h in the air and ultrasonically cleaned	annealed at 550 C for 2 h
Surface roughness parameters Ra or Sa	Sa cp-Ti: 0.297 µm Sa TNT: 0.22 µm No significant difference. *p* value not reported	Ra cp-Ti: 157 ± 41 nm Ra TNT: 235 ± 95 nm No significant difference: *p* value not reported	Ra cp-Ti: 8.9 ± 2.4 nm Ra TNT: 45.8 ± 6.3 nm *(p< 0.001)*	Ra cp-Ti: 9.5 ± 1.6 nm Ra TNT: 46.5 ± 2.8 nm No significant difference: *p* value not reported
Nanotubule diameter	an external and internal mean diameter of 119+/- 22 nm and 50+/- 11 nm, respectively.	80–100 nm in diameter and tube walls 15–20 nm thick.	100 nm and length of 1 μm	100 nm
Cell proliferation outcome	MTT assay Absorbance value Measured at 570 nm	MTS assay Absorbance value Measured at 490 nm	CCK-8 assay Absorbance value Measured at 450 nm	CCK-8 assay Absorbance value Measured at 450 nm
Cell proliferation timing	2 and 7 days	3, 4, 7 and 14 days	1, 3, 5 and 7 days	1, 3, 5 and 7 days
Protein expression outcome	ELISA Concentration (ng/ml/cell) Measured at OD 450 nm	ELISA Concentration (U/ml) Measured at OD 450 nm	ELISA Concentration (ng/ml) Measured at OD 450 nm	ELISA Concentration (ng/ml) Measured at OD 450 nm
Proteins assessed	COL-1	COL-1	COL-1 and FN	COL-1 and FN
Protein expression timing	6 hours, 2 days, and 7 days	3, 4, 7 and 14 days	1, 4 and 7 days	1, 3 and 6 days
Relative gene expression outcome	Not assessed	RT-qPCR Relative mRNA expression	RT-qPCR Relative mRNA expression	RT-qPCR Relative mRNA expression
Genes assessed	Not assessed	COL-1	COL-1, FN, ITGβ1 and VCL	COL-1, FN, ITGβ1 and VCL
Gene expression timing	Not assessed	3, 4, 7 and 14 days	4 and 24 hours	4 and 24 hours
Cell morphology evaluation	SEM	SEM	SEM	SEM
Cell morphology timing	6 hours	1, 3, 9 and 24 hours	1, 4 and 24 hours	1, 2, 4 and 24 hours
Statistical analysis	Wilcoxon Rank-Sum Median, LQ, UQ, min and max values	ANOVA Mean ± SD	ANOVA Mean ± SD	ANOVA Mean ± SD

*P.H.I* Primary healing implant, *ATCC* American Type Culture Collection, *TNTs* Titania nanotubes, *cp-Ti* Commercially pure titanium, *HGFs* Human Gingival Fibroblasts, *Sa* and *Ra* surface roughness parameters, *MTS* 3-(4,5-dimethylthiazol-2-yl)-5-(3-carboxymethoxyphenyl)-2-(4-sulfophenyl)-2H-tetrazolium, *MTT* 2-(4,5-Dimethyl-2-thiazolyl)-3,5-diphenyl-2H-tetrazolium bromide, *CCK-8* Cell Counting Kit-8; OD: Optical density, *ELISA* Enzyme-Linked Immunosorbent Assay, *SEM* Scanning Electron Microscopy, *RT-qPCR* Reverse Transcription- Quantitative real-time Polymerase Chain Reaction, *mRNA* messenger ribonucleic acid, *COL-1* Collagen Type 1, *FN* Fibronectin, *ITGβ1* Integrin β1, *VCL* Vinculin, *ANOVA* Analysis of variance, *SD* Standard deviation, *LQ* lower quartile, *UQ* upper quartile, *Min* minimum value, *Max* maximum value.

### Study characteristics

The publication dates ranged from 2013 to 2022. One study was carried out in Italy [43] and three in China [1, 25, 44]. Two studies were by the same group, Chen, and colleagues [1, 25]. The source of funding was acknowledged in all four studies. One study was done with titanium samples from a commercial organisation, Primary Healing Implant (P.H.I) company, in Italy [43]. Three studies received government funding - National Natural Science Foundation of China [1, 25, 44]. One study was supported by Natural Science Foundation of China for Young Scholars [44]. Three studies used the same commercial cell line of HGFs (HGF-1, CRL-2014), purchased from American Type Culture Collection (ATCC, Manassas, VA, USA) [1, 25, 44, 45]. The HGF-1, CRL-2014 cell line was isolated from the gingiva of a healthy 28-year-old, white male patient in 1989 [45]. One study used HGFs from biopsies, harvested from healthy human donors who underwent periodontal surgery, including a 35-year-old female and 56-year-old male patient [43]. The harvested cells were kept separate and not pooled [43]. The passage number varied between each study including 2-4 [43], 3-5 [44], 2-6 [25], and 3-6 [1].

For the titanium sample, two studies used sheets [1, 44], one study used plates [43] and the other used discs [25]. Smooth cp-Ti in two studies for comparison [1, 25]. Turned cp-Ti was used in one study [43] and polished cp-Ti in the other study [44]. The intervention in each study was TNTs fabricated using electrochemical anodization on a cp-Ti. Two surface roughness parameters were used Ra (nm) in three studies [1, 25, 44], and Sa (µm) in one study [43].

One study performed the tests twice in quadruple on both cell preparations (*n* = 8) [43]. One study performed all the experiments in triplicate [25]. Two studies measured cell proliferation by CCK-8 assay [1, 25]. One study used the MTT assay [43] and another study used the MTS assay [44]. Cell proliferation was quantified between 450-570 nm absorbance. Three studies measured cell proliferation up to 7 days [25, 43, 44] and one study up to 14 days [1].

Protein expression was quantified at 450 nm OD in all four studies [1, 25, 43, 44]. All studies measured the expression of COL-1 protein. Two studies also measured fibronectin protein expression [1, 25]. The timing of measuring protein expression outcome varied between 6 hours and 14 days.

Three studies measured relative gene expression using a two-step RT-qPCR process [1, 25, 44]. The relative gene expression of COL-1 was measured in three studies [1, 25, 44]. Other researchers also measured the relative gene expression of fibronectin (FN), vinculin (VCL) and integrin β1 (ITGβ1) [1, 25]. The timing of relative gene expression outcome measure varied from 4 hours and 14 days. SEM was used for cell morphology in all studies, with the timing varying between 1 to 24 hours.

For the statistical analysis, three studies used Analysis of Variance (ANOVA) and presented the data using mean ± standard deviation (SD) [1, 25, 44]. One study used Wilcoxon rank sum and presented the data using median, lower quartile (LQ), upper quartile (UQ), minimum (min) and maximum (max) values [43].

### Primary outcome measures

The primary outcome measures assessed are summarised in Table 4.

**Table 4:** Summary of Primary outcome measures

Outcome measure	Day	Guida et al. [43]	Liu et al. [44]	Chen and colleagues	
				Wang et al. [25]	Cao et al. [1]
Cell proliferation (Absorbance value)	3	Not assessed	cp-Ti: 0.97 ± 0.04 TNT: 0.96 ± 0.04 (*p>0.05*)	cp-Ti: 1.37 ± 0.1 TNT: 1.44 ± 0.13 No significant difference: *p* value not reported	cp-Ti: 0.28 ± 0.02 TNT: 0.29 ± 0.01 No significant difference: *p* value not reported
	7	cp-Ti: Median: 0.12 LQ: 0.106 UQ: 0.135 Min: 0.08 Max: 0.1 TNT Median: 0.43 LQ: 0.36 UQ: 0.45 Min: 0.34 Max: 0.57 (*p<0.001)*	cp-Ti:1.27 ± 0.02 TNT: 1.26 ± 0.02 No significant difference: *p* value not reported	cp-Ti: 2.11 ± 0.02 TNT: 2.37 ± 0.03 *(p<0.001)*	cp-Ti: 0.37 ± 0.03 TNT: 0.44 ± 0.04 *(p<0.05)*
	14	Not assessed	cp-Ti:1.41 ± 0.01 TNT: 1.48 ± 0.01 *(p<0.05)*	Not assessed	Not assessed
FN protein expression (Concentration value)	6	Not assessed	Not assessed	Not assessed	cp-Ti: 189 ± 3 ng/ml TNT: 239 ± 7 ng/ml *(p<0.05)*
	7	Not assessed	Not assessed	cp-Ti: 293.59 ± 9.85 ng/ml TNT: 329.06 ± 7.88 ng/ml *(p<0.01)*	Not assessed
COL-1 protein expression (Concentration value)	6	Not assessed	Not assessed	Not assessed	cp-Ti: 252 ± 12 ng/ml TNT: 278 ± 4 ng/ml *(p<0.05)*
	7	cp-Ti: Median: 17.25 ng/ml/cell LQ: 14.49 ng/ml/cell UQ: 17.67 ng/ml/cell Min: 13.71 ng/ml/cell Max: 18.59 ng/ml/cell TNT: Median 21.92 ng/ml/cell LQ: 21.06 ng/ml/cell UQ: 22.66 ng/ml/cell Min: 20.44 ng/ml/cell Max: 23.62 ng/ml/cell *(p<0.001)*	cp-Ti: 4.73 ± 0.03 U/ml TNT: 6.2 ± 0.1 U/ml *(p<0.05)*	cp-Ti: 299.99 ± 29.27 ng/ml TNT: 302.43 ± 21.96 ng/ml No significant difference: *p* value not reported Not assessed	Not assessed
	14	Not assessed	cp-Ti 5.97 ± 0.07 U/ml TNT 5.59 ± 0.14 U/ml *(P<0.05*).		Not assessed

*cp-Ti* Commercially pure titanium, *TNT* Titania nanotube, *COL-1* Collagen Type 1, *FN* Fibronectin, *LQ* lower quartile, *UQ* upper quartile, *Min* minimum value, *Max* maximum value.

#### Cell proliferation

All studies measured and plotted the mean absorbance against time duration. Three studies assessed the HGF cell proliferation at cp-Ti and TNT after 3 days, and none reported a statistically significant difference between the absorbance values [1, 25, 44]. All studies assessed the HGF cell proliferation at cp-Ti and TNT after 7 days, and three of the studies reported a significant difference with higher absorbance values for TNT compared to cp-Ti [1, 25, 43]. One study assessed the HGF cell proliferation between cp-Ti and TNT after 14 days and reported a significant difference with higher absorbance values for TNT compared to cp-Ti [44].

#### Protein expression

All studies plotted concentration against time. One study assessed the expression of fibronectin protein by HGFs at TNTs and cp-Ti after 6 days and reported a significant increase for TNTs compared to cp-Ti [1]. One study assessed the expression of fibronectin protein by HGFs at TNTs and cp-Ti after 7 days and reported a significant increase at TNTs compared to cp-Ti [25].

One study assessed the expression of COL-1 protein by HGFs at cp-Ti and TNTs after 6 days and reported a significant increase at TNTs compared to cp-Ti [1]. Three studies assessed the expression of COL-1 protein by HGFs at cp-Ti and TNTs after 7 days, and two of these studies reported a significant increase for TNTs compared to cp-Ti [43, 44]. One study assessed the expression of COL-1 protein by HGFs at cp-Ti and TNTs after 14 days and reported a significant increase at cp-Ti compared to TNTs [44]. One study did not report a significant difference in the expression of COL-1 protein by HGFs between cp-Ti and TNTs [25].

### Secondary outcome measures

The secondary outcome measures are shown in Table 5.

**Table 5:** Summary of Secondary outcome measures

Outcome measure	Time point	Guida et al. [43]	Liu et al. [44]	Chen and colleagues	
				Wang et al. [25]	Cao et al. [1]
Relative FN expression	24 hours	Not assessed	Not assessed	cp-Ti: 0.95 ± 0.15 TNT:1.72 ± 0.4 *(p<0.05)*	cp-Ti: 1.18 ± 0.19 TNT: 2.15 ± 0.05 *(p<0.05)*
Relative ITGβ1 gene expression	24 hours	Not assessed	Not assessed	cp-Ti: 0.97 ± 0.15 TNT: 1.69 ± 0.36 *(p<0.05)*	cp-Ti: 1.0 ± 0.2 TNT: 1.85 ± 0.14 *(p<0.05)*
Relative VCL gene expression	24 hours	Not assessed	Not assessed	cp:Ti: 0.94 ± 0.04 TNT: 1.21 ± 0.04 *(p<0.01)*	cp:Ti: 1.0 ± 0.5 TNT: 1.35 ± 0.07 *(p<0.05)*
Relative COL-1 gene expression	24 hours	Not assessed	Not assessed	cp-Ti: 0.97 ± 0.15 TNT: 1.38 ± 0.06 No significant difference; *p* value not reported	cp-Ti: 1.18 ± 0.13 TNT: 1.75 ± 0.05 *(p<0.05)*
	3 days	Not assessed	cp-Ti: 27.68 ± 1.67 TNT: 30.50 ± 6. *(p<0.05)*.	Not assessed	Not assessed
	4 days	Not assessed	cp-Ti 10.38 ± 3.34 TNT 35.11 ± 4.04 *(p<0.05)*.	Not assessed	Not assessed
	7 days	Not assessed	cp-Ti: 0.86 TNT: 35.29 ± 4.24 *(p<0.05)*	Not assessed	Not assessed
	14 days	Not assessed	cp-Ti: 4.44 ± 0.75 TNT: 10.43 ± 1.16 *(p<0.05)*	Not assessed	Not assessed
Cell morphology	1-24 hours	cp-Ti: Cells were spread out and spindle-like. No evidence of filopodia anchorage. TNT: Cell processes filopodia and lamellipodia formed intimate interaction with the TNT.	cp-Ti: Cells were spread out and spindle-like. No evidence of filopodia anchorage. TNT: Protruding pseudopodia, anchored to the TNT.	cp-Ti: Flat cells with little filopodia evident. TNT: Filopodia extending to the TNT.	cp-Ti: Disc shape. No evidence of filopodia anchorage. TNT: Protruding pseudopodia and filopodia attached to TNT.

*cp-Ti* Commercially pure titanium, *TNT* Titania nanotube, *FN* Fibronectin, *COL-1* Collagen Type 1, *ITGβ1* Integrin β1, *VCL* Vinculin.

#### Relative gene expression

Three studies reported relative mRNA expression by HGFs and plotted relative expression against time duration [1, 25, 44]. Two studies specified the use of 2-delta delta cycle threshold method (2^−∆∆Ct^) [1, 44]. Two studies reported a significant increase in the relative gene expression of fibronectin, integrin β1 and vinculin at TNTs compared to cp-Ti [1, 25]. They also assessed the relative gene expression of COL-1 after 24 hours and one of these studies reported a significant increase at TNTs compared to cp-Ti [1]. One study showed a significant increase in the relative gene expression of COL-1 at TNTs compared to cp-Ti after each time point of 3, 4, 7 and 14 days [44].

#### Cell morphology

HGFs on cp-Ti were spread out and spindle-like [43, 44]. There was little or no evidence of cell extensions or filopodia from HGFs on cp-Ti. Three studies reported that the cell extensions from HGFs protruded and attached onto the TNT surface [1, 25, 44]. One study reported that the cell extensions from HGFs formed an intimate interaction with the TNT surface [43].

#### Trends in relative gene expression and protein expression

A significant increase in the relative gene expression and protein expression of fibronectin at TNTs compared to cp-Ti was shown [1, 25]. A significant increase in the relative gene expression and protein expression of COL-1 at TNTs compared to cp-Ti was also reported [1, 44].

#### Quality of the studies included in this review

The risk of bias was assessed, and each outcome measured separately, (Tables 6, 7, 8 and 9). All the studies had a *“probably high risk of bias”* or *“not reported”* for most criteria. One study used primary cell cultures harvested from one male and one female donor and did not report on randomization nor allocation concealment [43]. Three studies used a homogenous cell line, as such a “probably low risk of bias” rating was assigned for randomization and allocation concealment [1, 25, 44]. Three studies received a commercial cell line previously tested for mycoplasma [1, 25, 44]. In all the included studies, the researchers were not blinded during the experiment nor outcome assessment, risking biased results [1, 25, 43, 44]. None of the included studies reported incomplete outcome data through unexplained plate or well loss. All the included studies used gold standard and widely accepted methods to assess the outcomes including colorimetric cell proliferation assays, ELISA, RT-qPCR and SEM. Three studies used the ANOVA [1, 25, 44]. This parametric test assumes normally distributed data and homogeneity of variance [46, 47]. Two of the studies did not confirm normality nor homogeneity [1, 25]. Only one of these studies reported testing for homogeneity of variance and stated cases displaying heterogeneity [44]. The potentially incorrect use of the parametric tests, risked biased results. OHAT [48, 49] reported that studies *with “probably high risk of bias”* or “*definitely high risk of bias”* should not be excluded from the overall evidence, as this would reduce the evidence base available for an evaluation. The heterogeneity across the studies including characteristics, timings of observations, outcomes, statistical methods precluded a meta-analysis.

**Table 6:** Risk of bias rating for cell proliferation [48, 49]

Risk of Bias Criteria	Guida et al. [43]	Liu et al. [44]	Chen and colleagues	
			Wang et al. [25]	Cao et al. [1]
Randomisation	NR	+	+	+
Allocation concealment	NR	+	+	+
Identical experimental conditions	−	+	NR	NR
Blinding of researchers during study	NR	NR	NR	NR
Incomplete outcome data	NR	NR	NR	NR
Exposure characterisation	+	++	++	+
Blinding of outcome assessors	NR	NR	NR	NR
Outcome reporting	NR	NR	NR	NR
Other sources of bias	−	−	−	−

**Table 7:** Risk of bias rating for protein expression [48, 49]

Risk of Bias Criteria	Guida et al. [43]	Liu et al. [44]	Chen and colleagues	
			Wang et al. [25]	Cao et al. [1]
Randomisation	NR	+	+	+
Allocation concealment	NR	+	+	+
Identical experimental conditions	NR	+	NR	NR
Blinding of researchers during study	NR	NR	NR	NR
Incomplete outcome data	NR	NR	NR	NR
Exposure characterisation	+	++	++	+
Blinding of outcome assessors	NR	NR	NR	NR
Outcome reporting	+	NR	+	NR
Other sources of bias	**−**	**−**	**−**	**−**

**Table 8:** Risk of bias rating for relative gene expression [48, 49]

Risk of Bias Criteria	Guida et al. [43]	Liu et al. [44]	Chen and colleagues	
			Wang et al. [25]	Cao et al. [1]
Randomisation	Outcome not assessed	+	+	+
Allocation Concealment	Outcome not assessed	+	+	+
Identical experimental conditions	Outcome not assessed	+	NR	NR
Blinding of researchers during study	Outcome not assessed	NR	NR	NR
Incomplete outcome data	Outcome not assessed	NR	NR	NR
Exposure characterisation	Outcome not assessed	++	++	NR
Blinding of outcome assessors	Outcome not assessed	NR	NR	NR
Outcome reporting	Outcome not assessed	NR	NR	NR
Other sources of bias	Outcome not assessed	−	−	−

**Table 9:** Risk of bias rating for cell morphology [48, 49]

Risk of Bias Criteria	Guida et al. [43]	Liu et al. [44]	Chen and colleagues	
			Wang et al. [25]	Cao et al. [1]
Randomisation	NR	+	+	+
Allocation concealment	NR	+	+	+
Identical experimental conditions	−	−	−	−
Blinding of researchers during study	NR	NR	NR	NR
Incomplete outcome data	NR	NR	NR	NR
Exposure characterisation	+	++	++	+
Blinding of outcome assessors	NR	NR	NR	NR
Outcome reporting	−	−	−	−
Other sources of bias	−	−	−	−

## Discussion

This systematic review showed that HGFs have an enhanced soft tissue attachment to TNTs compared to cp-Ti or Ti-Al6-V4, in-vitro. The results seem to indicate that HGFs have an enhanced contact guidance to TNTs compared to cp-Ti. The HGFs change their orientation and appear to favour the TNT surface compared to cp-Ti, though a definitive biological attachment to the TNTs was not demonstrated.

Enhanced cell proliferation and increased production of adhesion related genes and proteins at TNTs compared to cp-Ti has also been demonstrated [7, 19]. The resulting higher surface area of TNTs may enable the deposition of more collagen fibres and fibronectin [19, 22, 25].

Other researchers have also identified that HGFs displayed many filopodia and lamellipodia extensions, anchored onto the TNTs [7, 13]. These cellular extensions can guide the HGFs to enhance the activation and translation of adhesion related genes and proteins and promote the formation of focal adhesions e.g., integrin α5β1 and α1β1 [13, 29]. Chen and colleagues reported increased relative gene expression of integrin β1 by HGFs at TNTs. Proteins such as fibronectin bind on the extracellular side of the focal adhesion, and an actin filament will interact with the intracellular side, permitting signal transduction and various processes including cell morphology, migration, proliferation, and adhesion [50, 51]. Moon et al. [52] reported that vinculin may be an important protein related to the strength of cell attachment to the surface. Numerous adhesions with enhanced vinculin were identified on TNTs. The enhanced attachment of HGFs to TNTs may be due to the increased expression of integrins and vinculin [1, 7, 8, 25]. Xu et al. used a micro-rough titanium for comparison, reducing the general applicability to the human population, as a smooth surface is more pertinent for the soft-tissue-implant interface [9, 53].

Various surface treatments have been proposed to increase the surface activity of the dental implants to initiate cell motility to strengthen osseointegration. A review by Pesce et al., on the effects of UV and non thermal plasma functionalization of dental titanium implants on the osseointegration shows increased osteoblast migration [54]. Research by Canullo et al. on argon plasma treated healing abutment surfaces showed reduction in bacterial microbiome, biofilm formation and soft tissue inflammation [55] and vacuum plasma treated implants confirm the effectiveness of plasma treatments on cell adhesion and fibroblast activity [56]. Enhancing the wettability of the surface of the healing abutments through biofunctionalization methods boosts surface free energy, which inversely affects the presence of contaminants. Clinically, this increased wettability promotes stronger fibroblast adhesion, even in the early healing stages. This is evidenced by the formation of filopodia extensions and improved tissue organization, with denser collagen fibers and more oblique fibers [55].

Interestingly, no significant difference in cell proliferation between TNTs and polished titanium was reported initially [2]. Furthermore, Guo et al. showed a significantly increased cell proliferation at the polished titanium alloy compared to TNTs [13]. It has been postulated that cell proliferation at TNTs may be reduced due to contact inhibition causing cells to become compromised due to lack of absorption of carbohydrates, amino acids, vitamins, minerals, hormones, and growth factors [13]. The accumulation of toxic metabolites in the culture medium may also lead to low activity or cell death [13, 57, 58]. Guo et al. reported significantly higher expression of fibronectin protein after 4 hours and 1 day at the polished titanium alloy compared to TNTs inferring reduced functionality of HGFs at TNTs [13].

The quantification of soft tissue attachment is highly challenging. The outcomes were measured at different times, making comparisons between groups very difficult. The observation time ranged between 1 hour to 14 days. The relevance and applicability of this remains questionable as the soft-tissue attachment takes approximately 4-6 weeks to form and 6-8 weeks to mature [59].

One study used the same time duration for studying both relative gene and protein expressions, enabling comparisons between the outcomes [44]. One study measured protein expression at 1, 4 and 7 days [25]. One study measured protein expression at 1, 3 and 6 days [1]. Two studies measured relative gene expression at 4 and 24 hours only, which restricted comparisons between the relative gene expression and protein expression outcomes [1, 25].

The inclusion and exclusion criteria were appropriate to address the research question. Unlike previous systematic reviews in this field which have included numerous materials for the intervention [27, 32, 33], this review presents results specifically related to TNTs, making these studies readily comparable.

### Limitations of this review

A prominent limitation of this systematic review, was the availability of a sole researcher (SB) for screening, study selection, data extraction, risk of bias assessment, appraisal, and synthesis. The researchers (SB, AS, MK, NC, WA, and FB) all contributed to the write-up. It is best practice for two people to independently carry out screening, study selection, data extraction and assessment of methodological quality/risk of bias [60, 61]. However, this was not possible due to the various restrictions and increases the risk of bias and errors [61]. Single data extraction may cause more errors than data extraction carried out by two reviewers [62]. However, this did not substantially influence the estimates of treatment effects [62]. Errors in data extraction had little impact on any review conclusions [63].

The literature obtained was limited, with two of the included studies from the same author group. This review encompassed a new area of research with a limited evidence base. To compensate for limited results from the electronic searches, supplementary hand searching and screening of the reference lists of all full-text studies obtained, and previous systematic reviews was carried out to improve the sensitivity. However, this review included published literature and did not include grey literature which risks the introduction of publication bias, as some information could have been missed [64]. The possible variations in publication bias including non-English language publication, grey literature and un-published studies, risk overestimating the effect size [61, 64].

A variety of electronic databases were utilised, including a dental specific database, DOSS, to identify the highest proportion of relevant studies. However, this review excluded non-English studies. This may be a limitation, as vital information published in other languages could have been missed, risking language bias [65]. However, a systematic review by Morrison et al. concluded no evidence of bias when language restrictions were applied [66].

Among the Risk assessment tools recommended by NHMRC for invitro studies, the authors found OHAT tool to be suitable for this review. Other popular tools like Cochrane Collaboration, Joanna Briggs Institute Clinical Appraisal Checklist for Experimental Studies and Timmer’s Analysis Tool would have provided better quality of evidence.

The risk of bias suggests that the overall quality of the evidence is uncertain [67]. The assessment of the risk of bias regarding the methods used such as randomisation and allocation concealment, are difficult to apply to in-vitro studies and may, infact not be required when a homogenous cell line is utilised [68]. For the identical experimental conditions and outcome reporting criteria, most of the included studies were of *“probably high risk of bias”* or *“not reported.”*

Across the outcomes, the 95% confidence interval excluded no effect. There is reasonable confidence that an effect is likely to be present [60, 67]. In-vitro studies do not usually include a sample size calculation, instead use non-parametric statistical tests for data analysis [69]. Only one study used a non-parametric test [43]; however, the inferences from non-parametric tests are lower compared to parametric tests [46, 47]. Other research used parametric tests and did not confirm normality nor homogeneity of variance, thus limiting the effectiveness of the tests [1, 25, 44].

One study used HGFs from biopsies, harvested from two healthy human donors including a 35-year-old female and 56-year-old male patient [43]. The harvested cells were kept separate for each experiment. However, by pooling the data of both donor groups into the analysis, they failed to consider individual variabilities such as age, sex, race, medical history, smoking status, alcohol habits, periodontal health, local mucosal condition, healing time and batch-to-batch variations. This can lead to variation in the behaviour of the HGFs risking misleading results. Primary cell cultures often represent a heterogenous population, particularly as they are in a constant state of differentiation [70, 71]. Other studies used the same commercial cell line of HGFs (HGF-1, CRL-2014) purchased from the cell bank [1, 25, 44]. ATCC confirmed the source was a healthy patient [45]. However, the possibility of mutations or oncogenes in the cell lines were not considered. These can interfere with the cellular phenotype, risking confounding factors which may threaten the internal validity of the results [27, 57].

The studies included in this review exclusively assessed HGFs, which are a more representative sample to the connective tissue. However, the population were a single cell type cultured in a monolayer which cannot be applied to the general population, threatening the external validity of the results. However, at times in-vitro studies cannot be translated to the clinical situation as it does not duplicate the human physiology which includes a multi-layered process, micro-environment with an abundance of different cell types interacting with one another, polarised cell phenotypes, host inflammatory response, mechanical loading, etc [22, 72, 73]. Therefore, a direct applicability to the human population is highly limited.

The terms *“smooth,” “turned,”* and *“polished”* used to define the cp-Ti comparator surface introduced heterogeneity between the studies. None of the studies included specified the grade of cp-Ti utilised. Given that only cp-Ti (grade IV) or Ti-Al6-V4 alloy (grade V) are used to produce abutments, this poses doubt on the generalisability and external validity of the findings [27]. Furthermore, the use of plates, sheets and discs for the cp-Ti samples may limit the translatability to the implant abutment surface utilised in the human population.

### Limitations of TNTs

A potential consequence of nano-engineered titanium implants is biofilm formation and bacterial colonisation due to the increased surface roughness [33]. Bacterial colonisation and penetration through the soft tissue-implant interface, risks peri-mucositis, peri-implantitis and implant failure [3, 9, 28, 74, 75]. A threshold surface roughness of Ra 0.2 µm has been reported [76]. No further changes in bacterial adhesion can be detected below the threshold. One study reported the mean surface roughness of TNTs of Ra 0.235 µm, suggesting an increased risk of bacterial adhesion [44]. The mean surface roughness of TNTs was lower than the 0.2 µm threshold, reducing the risk of bacterial adhesion [1, 25]. One study utilised a ‘Sa’ as a surface roughness parameter, thus, this threshold is not applicable [43]. Other groups have reported increased bacterial adhesion at TNTs compared to conventional titanium possibly due to the amorphous phase of TNTs and residual fluorine on the TNTs [77, 78]. Future research is needed to assess the TNTs influence on the bacterial adhesion, including the bacterial count, composition, and strength of biofilms [79–81].

There was no evidence of cytotoxicity for TNTs as cell proliferation of HGFs occurred at these surfaces. However, nano-engineered surfaces may challenge the host immune system [53]. The stability of the implant in load-bearing environments may be at risk, causing cracks and delamination of the TNTs [17, 22, 53]. This can lead to leaching and release of titanium dioxide nano- or micro-particles or titanium, aluminium, or vanadium ions into the surrounding tissues which may elicit an immuno-inflammatory response [17, 22, 82, 83]. Research on the effects of TNTs delamination, fracture, and particles release on immune response are required.

This research may be useful for researchers, clinicians, patients, and manufacturers. The applications of TNTs show promise. However, the knowledge gaps may hinder their clinical translation

## Conclusions

This systematic review is limited; however, it can be concluded that HGFs give an enhanced contact guidance to TNTs compared to cp-Ti. More work is needed on the mechanical integration of collagen with the substrate surface to whether biological attachment has occurred. Future research should also consider the methodological limitations of published in-vitro studies. A long-term in-vivo animal design with physiological fluids and load-bearing conditions would be ideal to help bridge the gap between the laboratory and clinic.

## Supplementary information

Index 1

PRISMA Checklist
