# Supplementary material for: Soft tissue attachment of human gingival fibroblasts to titanium dioxide nanotubes compared to commercially pure titanium and its alloys: a systematic review
Source: BDJ Open. 2025 Jun 18;11:58. doi: 10.1038/s41405-025-00293-0 (PMC12177073; doi:10.1038/s41405-025-00293-0)
Supplement: Supplementary file 1 — Index 1 [file 41405_2025_293_MOESM1_ESM.pdf]

Index 1: ***Search strategy for MEDLINE (Host: EBSCO)***

Main concept	Search ID#	Search terms
Human gingival fibroblasts	S1	Human gingival fibroblast*
	S2	HGF
	S3	HGF-1
	S4	(MH "Fibroblasts")
	S5	(MH "Connective Tissue Cells")
	S6	S1 OR S2 OR S3 OR S4 OR S5
Titanium dioxide nanotubes	S7	Nanotube
	S8	Titanium nanotube
	S9	Titanium dioxide nanotube
	S10	TiO2 nanotube
	S11	TDNT
	S12	TNT
	S13	Nanostructured ti* surface*
	S14	Nano-structured ti* surface*
	S15	Surface modification
	S16	Modified surface*
	S17	(MH "Nanotubes")
	S18	(MH "Nanomedicine")
	S19	(MH "Surface Properties")
	S20	(MH "Electrochemistry")
	S21	(MH "Nanotechnology")
	S22	S7 OR S8 OR S9 OR S10 OR S11 OR S12 OR S13 OR S14 OR S15 OR S16 OR S17 OR S18 OR S19 OR S20 OR S21
Commercially pure titanium or titanium alloy	S23	Commercially pure ti*
	S24	cpti
	S25	Machined
	S26	Machined surface
	S27	Machined ti*
	S28	Smooth
	S29	Smooth surface
	S30	Smooth ti*
	S31	Polished
	S33	Polished surface
	S33	Polished ti*
	S34	Turned
	S35	Turned surface
	S36	Turned ti*
	S37	Dental implant*
	S38	(MH "Titanium")
	S39	(MH "Dental Implants")
	S40	(MH “Dental Implant-Abutment Design)
	S41	(MH “Dental Abutments”)

	S42	S23 OR S24 OR S25 OR S26 OR S27 OR S28 OR S29 OR S30 OR S31 OR S32 OR S33 OR S34 OR S35 OR S36 OR S37 OR S38 OR S39 OR S40 OR S41
Soft tissue attachment	S43	Adhesion
	S44	Proliferation
	S45	Attachment
	S46	Soft tissue attachment
	S47	Protein expression
	S48	Cell adhesion
	S49	Cell proliferation
	S50	(MH “Collagen Type 1”)
	S51	DE "PROTEIN expression"
	S52	S43 OR S44 OR S45 OR S46 OR S47 OR S48 OR S49 OR S50 OR S51
	S53	S6 AND S22 AND S42 AND S52
**Limiters** - Date of Publication: 2012-2022	S54	267
